# Prescription patterns and appropriateness of NSAID therapy according to gastrointestinal risk and cardiovascular history in patients with diagnoses of osteoarthritis

**DOI:** 10.1186/1741-7015-9-38

**Published:** 2011-04-14

**Authors:** Angel Lanas, Guillermo Garcia-Tell, Beatriz Armada, Angel Oteo-Alvaro

**Affiliations:** 1 University of Zaragoza Medical School, Aragón Health Research Institute (IIS Aragón), CIBERehd, Zaragoza, Spain; 2 CS Salvador Pau, Assistant Professor Department of Medicine.,University of Valencia, Spain; 3 Pfizer S.L.U., Madrid, Spain; 4 Service of Orthopedics, Hospital Gregorio Marañon, Madrid, Spain

## Abstract

**Background:**

Prescription of non-steroidal anti-inflammatory drugs (NSAIDs) should be based on the assessment of both gastrointestinal (GI) and cardiovascular (CV) risk for the individual patient. We aimed to assess the GI/CV risk profile and the pharmacological management of patients with osteoarthritis (OA) in clinical practice.

**Methods:**

We conducted a cross-sectional, multicentre, observational study of consecutive OA patients that visited 1,760 doctors throughout the Spanish National Health System (NHS) in a single day. The presence of GI risk factors, CV histories, hypertension and current pharmacological treatments was recorded.

**Results:**

Of the 60,868 patients, 17,105 had a diagnosis of OA and were evaluable. The majority (93.4%) had more than one GI risk factor and 60.3% were defined to be at high-GI risk. Thirty-two percent had a history of CV events, 57.6% were treated with anti-hypertensive therapy and 22.6% had uncontrolled hypertension. One-fifth of patients were treated with non-NSAID therapies, whereas the remaining patients received NSAIDs. Non-selective NSAIDs (nsNSAID) plus proton pump inhibitor (PPI) or cyclooxigenase-2 (COX-2)-selective NSAIDs alone were more frequently prescribed in patients at increased GI risk. Patients with a positive CV history received nsNSAIDs or COX-2-selective NSAIDs in 41.3% and 31.7% of cases, respectively. When both the GI and CV histories were combined, 51% of the overall population was being prescribed drugs that were either not recommended or contraindicated.

**Conclusions:**

Over 90% of patients with OA are at increased GI and/or CV risk. In over half of these patients, the prescription of NSAIDs was not in accordance with current guidelines or recommendations made by regulatory agencies.

## Background

Therapeutic intervention in osteoarthritis (OA) is focused on reducing pain and improving functional activity [1,2]. Among pharmacological treatments, NSAIDs are commonly prescribed because of their clinical efficacy, despite the well-known gastrointestinal (GI) side effects associated with their use [3]. Cyclooxigenase-2 (COX-2)-selective NSAIDs were developed to diminish the GI adverse events caused by non-selective NSAIDs (nsNSAIDs) [4]. COX-2-selective and most nsNSAIDs can be associated with increased cardiovascular (CV) risk, which has prompted the necessity for assessments of both GI and CV risk in patients who need these medications [5-8]. All NSAIDs are also associated with other side effects, including hypertension, water retention, heart failure and renal insufficiency [9].

Based on these findings, the Food and Drug Administration (FDA) [10], the European Medicines Agency (EMA) [11], and different scientific societies [12-14] agree that the medical management of patients who require NSAIDs must be based upon the previous assessment of GI and CV risk factors in the individual patient. Guidelines recommend the use of nsNSAIDs plus a gastroprotectant or a COX-2-selective NSAID alone in patients with one or more GI risk factors [15,16]. A COX-2-selective agent plus a proton pump inhibitor (PPI) is recommended in those with the highest GI risk, whereas nsNSAIDs and COX-2-selective inhibitors should be avoided in patients with high GI and CV risks [11-16]. The FDA clearly states that CV risk is associated with all NSAIDs, excluding aspirin [10]. The EMA contraindicates the use of the COX-2-selective agents in patients with previous CV events and establishes that adverse CV events with the use of nsNSAIDs cannot be excluded. In addition, the EMA contraindicates the use of etoricoxib in the presence of uncontrolled hypertension [11].

It is unknown whether these recommendations made by regulatory agencies and guidelines are being followed. Therefore, our study was aimed at evaluating the GI and CV risk profiles in patients with OA as well as the pharmacological management and the appropriateness of therapies based on current recommendations issued by European guidelines and the European Medicines Agency [11,12,14,16].

## Methods

### Sample and design

This was a cross-sectional, multicenter, observational study in OA patients who were considered candidates for NSAID medication. All data were collected in one single day (25 March 2009) in all participating centers. A total of 1,760 investigators from the Spanish National Health System representing the two specialties that most frequently treat patients with OA in our country's primary care physicians and orthopedists visited a total of 60,868 consecutive patients seen in this one day. The investigators registered the total number of patients who visited their medical centers on 25 March 2009. Patients included in the study had to meet the following criteria: 1) male or female aged over 18 years, 2) established diagnosis of osteoarthritis in medical records, 3) informed verbal consent. Patients received drug treatment and/or medical care according to the usual clinical habits of the attending doctor.

### Variables related to GI and CV risk classification

The following well known GI risk factors were assessed [12-17]: age 65 or older; concomitant acetyl salicylic acid (ASA), corticosteroid, or anticoagulant use; previous history of ulcers, ulcer bleeding, or dyspepsia; and the use of two NSAIDs or a high dose of one NSAID (a dose was considered high if the NSAID was prescribed at the maximum dose recommended for the symptomatic treatment of arthritis pain, as described elsewhere [17]. CV low dose ASA was considered any dose 300 mg/day or less. *Helicobacter pylori *infection was not considered because the proportion of patients from whom this information was available was very low

For the purposes of this study and based on previous reports [12-17], we classified patients into three GI categories. Patients at low GI risk were considered to be those without any of the above-mentioned clinical conditions. Patients at moderate GI risk included those with at least one of the following GI risk factors: 1) age 65 or older, 2) concomitant use of low-dose ASA, 3) concomitant use of corticosteroids, 4) history of symptomatic peptic ulcer, 5) history of dyspepsia, 6) current high-dose NSAID use and, 7) current use of two NSAIDs. As described elsewhere [17], patients at high GI risk were those with either a GI bleeding history, concomitant use of NSAIDs and anticoagulants or the presence of three risk factors of those described for moderate GI risk or any of those factors combined with an uncomplicated peptic ulcer. These combinations were based on the estimated incidence of events obtained from combining different risk factors that would put patients at a similar risk level to those with a history of bleeding peptic ulcers [18,19]. In this way, in low GI risk patients, the expected rate of upper GI complications should not exceed 1.5 events per 100 patients per year, whereas for those at moderate GI risk, the rate should be between 1.5 to 10, and for high GI risk patients, the rate should be greater than 10 events per 100 patients per year. Patients being treated with anticoagulants (warfarin or coumadin) and NSAIDs were considered high risk, because bleeding events in anticoagulated patients can be more severe [20].

In addition to GI risk factors, CV data were also collected which, according to recommendations of different government agencies and the EMA, limit the use of some NSAIDs. Information regarding previous CV history including myocardial infarction, angina, stroke, peripheral arteriopathy or heart failure, was collected. These diagnoses were confirmed by examination of patient medical records. The presence of hypertension history and anti-hypertensive drug therapy was also collected. Blood pressure levels were obtained at the clinical visit and compared with previous values recorded in patient charts, if available. Uncontrolled hypertension was considered if the systolic blood pressure was greater than 140 mmHg or the diastolic blood pressure greater than 90 mmHg at the medical visit.

Current OA medications taken by patients and prescriptions made at the clinical visit were also recorded as well as the type of NSAIDs and gastroprotective agents used. COX-2-selective agents included celecoxib or etoricoxib, whereas all other prescribed NSAIDs were considered non-selective.

### Statistical analysis

The descriptive analysis of the patients included demographic, clinical characteristics, medical history and pharmacological treatments. Quantitative and qualitative variables were analyzed using measurements of central tendency (mean, median) and measurements of dispersion (95% confidence interval). Furthermore, qualitative variables were defined according to their absolute and relative frequencies. The mean and SD are reported for continuous variables and percentages for qualitative variables. Student's *t*-test was used to analyze continuous variables and the Chi-square test for quantitative variables. Tests were two-tailed with a significance level of 5%. Data were analyzed with SAS 8.2 statistical software (SAS Institute, Cary, NC, USA). Due to a problem with the hard copy of the CRF, gender data were not collected and, therefore, cannot be provided.

#### Sample size

At least 5,000 patients who met the selection criteria were estimated for inclusion in the study. This sample size would allow the estimation of the percentage of patients with the presence of cardiovascular history (yes/no) and gastrointestinal risk factors (yes/no), with a significant level of 95% and a precision 1.5% in a two-sided test, assuming a population proportion in the worst case (P = Q = 50%). A replacement rate of 15% was estimated. A sample size of 17,000 patients covered this estimate with an accuracy of 0.8%.

### Ethical considerations

The study complied with all ethical considerations involving human subjects, as adopted in the 18^th ^World Medical Assembly, Helsinki, Finland. All information recorded was obtained following standard clinical guidelines, and patients were not subjected to any therapeutic or diagnostic experimentation. The study followed standard security and confidentiality measures, complying fully with Spanish legislation regarding data protection (Ley Orgánica de 15/99). The work was presented to and approved by the Regional Ethics Committee for Clinical Research, Hospital Clinic de Barcelona (registration number 2009/4747). The use of identification numbers, instead of other patient identifiers, ensured patient names remained confidential.

## Results

### Characteristics of patients

From a total of 60,868 patients seen by the 1,760 participating investigators in one day, 21,448 patients suffered from musculoskeletal diseases, but eventually 4,343 were excluded from the analysis because it was unclear whether the underlying disease was OA, leaving a final study sample of 17,105 patients.

#### Gastrointestinal risk

**Figure 1:**
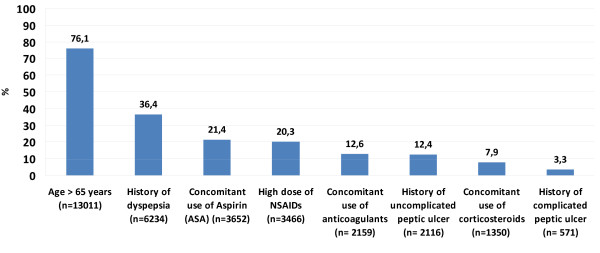
**GI risk factors in the study population (N = 17,105)**. Percentage of patients with each GI risk factor.

**Figure 2:**
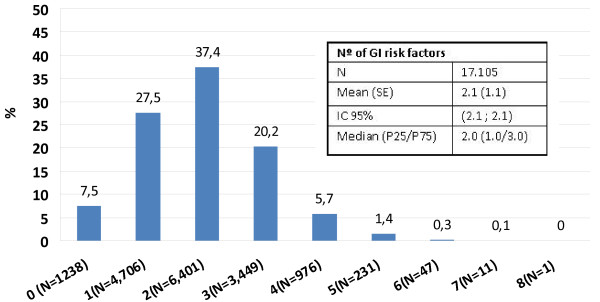
**Number of GI risk factors by number of patients (N = 17,105)**.

Figures 1 and 2 summarize the GI risk factors found in the study population. An age of 65 or older was most common (76.1%), whereas a history of complicated ulcers was the least common risk factor (3.3%). Overall, only 6.6% of patients had no GI risk factors, whereas the rest had at least one, and 65% had two or more. Based on these risk factors, >90% of OA patients were at increased GI risk and 60.3% of them could be considered at high risk for GI events.

#### Cardiovascular history

A history of previous CV events and uncontrolled hypertension are the two CV factors that limit the use of COX-2-selective agents and nsNSAIDs according to regulatory agencies. Of 16,470 patients with recorded CV data, 31.9% (5,256) had a history of at least one previous CV event; 22.6% (3,647/16,157) had uncontrolled hypertension and 57.6% (9,540/16,226) were taking anti-hypertensive drugs. Among those with a history of CV events, 2,064/5,256 (39.3%) were taking low-dose ASA.

**Figure 3:**
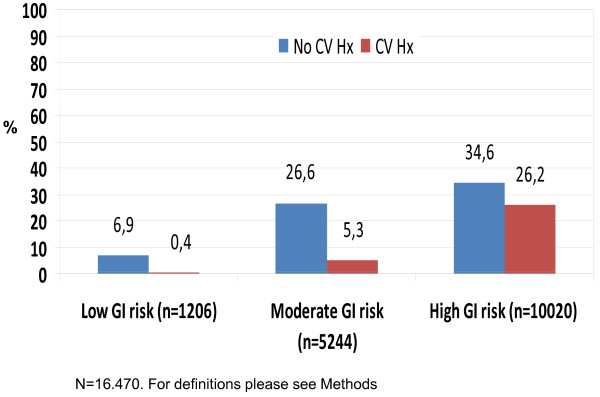
**Gastrointestinal risk and CV history in the OA study population**.

An association between GI and CV risk was observed. More patients with a positive CV history (Figure 3) or receiving antihypertensive therapy (71.4% of 9,354 patients) pertained to the high GI risk group than to the moderate or no GI risk groups.

### Pharmacological treatment

The mean number of OA medications prescribed to patients for underlying OA was 1.9 ± 0.9, and 93.9% of patients were receiving between one and three treatments. Of 17,105 patients with information regarding OA therapy, 20.4% were prescribed non-NSAID therapies including paracetamol (alone or combined with opioids or symptomatic slow acting drugs, including glucosamine, chondroitin sulfate, and diacerein (SYSADOAs) in 18.2% of cases. NsNSAIDs were prescribed to 46.3% of patients, and COX-2-selective inhibitors to 32.7%.

### Appropriateness of drug use in OA patients based on the GI and CV risk

Table 1 summarizes drug therapy according to GI risk. Non-selective NSAIDs were similarly prescribed in the three GI risk levels, although gastroprotective therapy and COX-2-selective inhibitors were more frequently prescribed to those at increased GI risk (*P *< 0·0001).

**Table 1:** Type of prescription according to GI risk

Type of drug	Low GI risk N (%)	Moderate GI risk N (%)	High GI risk N (%)	*P*-value**
	N = 1283	N = 5511	N = 10311	
**SYSADOA**	55 (4.3)	120 (2.2)	80 (0.8)	**<0.0001**
**Paracetamol**	130 (10.1)	535 (9.7)	841 (8.2)	**0.001**
**Opioids**	12 (0.9)	55 (1.0)	59 (0.6)	**0.008**
Combinations of any of the 3*	80 (6.2)	424 (7.7)	1103 (10.3)	**<0.0001**
**NsNSAIDs**	615 (47.9)	2,567 (46.6)	4,734 (45.9)	**0.337**
nsNSAID+ GP***	*344/615 (55.9*)	*2,093/2,567 (81.5)*	*4,367/4,734 (92.2)*	***<0.0001***

**Cox-2 NSAID**	294 (22.9)	1,636 (29.7)	3,669 (35.6)	**<0.0001**
Cox-2 + GP***	*96/294 (32.7)*	*880/1,636 (53.8)*	*2.800/3,669 (76.3)*	***<0.0001***

CV HX = cardiovascular history; GP = gastroprotectant (>91% proton pump inhibitors).

* Sysadoa, paracetamol, opioids; ** *P-*values denotes differences among groups overall; *** nsNSAIDS + GP data express the number of patients and % of patients with nsNSAIDs prescription that also take a gastroprotective agent. Also, COX-2 + GP data express the number of patients and % of patients with COX-2 selective inhibitors prescriptions that also take a gastroprotective agent.

Concerning the type of drugs prescribed according to CV risk, nsNSAIDs were more frequently prescribed to those with no history of CV events (5,452/11,214 = 48.6%) when compared to those with a previous history of CV events (2,169/5,256 = 41.3%) (*P *< 0·0001). Over one-third of patients with and without CV history received a COX-2-selective NSAID (33.5% vs. 31.7%; *P *= 0·02, respectively). Of 3,647 patients with uncontrolled hypertension, 82% were receiving NSAIDs (nsNSAIDs = 48.4% or COX-2-selective = 33.6%). In 10.7% of these cases, etoricoxib was the prescribed drug. The prescription of naproxen was rather small (3.4% of patients; 586 of 17,105). Of all patients treated with naproxen, 31.1% (175/562) had a previous CV history, 27.5% (155/563) had uncontrolled hypertension, and 54.7% (306/559) were taking antihypertensive treatment.

Within the overall OA population of this study, those patients with high GI risk and CV history represents 25.3% of the total. As shown in Table 2, 74.4% of this subpopulation (3,217/4,323 patients) received nsNSAIDs or COX-2-selective NSAIDs, which is considered inappropriate or contraindicated therapy for them, according to current guidelines [11-16].

**Table 2:** Type of prescription based on GI risk and CV history

Prescription	Low GI risk		Mod. GI risk		High GI risk		P** value
	No CV Hx*	CV Hx	No CV Hx	CV Hx	No CV Hx	CV Hx	
	N (%)	N (%)	N (%)	N (%)	N (%)	N (%)	
	N = 1,144	N = 62	N = 4,373	N = 871	N = 5,697	N = 4,323	
**SYSADOA**	52 (4.6)	0 (0.0)	92 (2.1)	22 (2.5)	44 (0.8)	33 (0.8)	**<0.0001**
**Paracetamol**	108 (9.4)	13 (21.0)	391 (8.9)	121 (13.9)	331 (5.8)	480 (11.1)	**<0.0001**
**Opioids**	11 (1.0)	1 (1.6)	40 (0.9)	11 (1.3)	28 (0.5)	28 (0.7)	**0.03**
Combinations of any of the 3*	72 (6.2)	5 (8.0)	306 (7.0)	97 (11.1)	498 (8.7)	586 (13.5)	**<0.0001**
**nsNSAIDs**	554 (48.4)	27 (43.5)	2,091 (47.8)	353 (40.5)	2,807 (49.3)	1,789 (41.4)	**<0.0001**
nsNSAID+ GP***	*307/554 (55.4)*	*18/27 (66.7)*	*1,687/2,091 (80.7)*	*302/353 (85.6)*	*2,552/2,807 (90.9)*	*1,691/1,789 (94.5)*	***<0.0001***
**Cox-2 NSAID**	267 (23.3)	13 (21.0)	1,335 (30.5)	227 (26.1)	2,154 (37.8)	1,428 (33.0)	**<0.0001**
Cox-2 + GP***	*89/267 (33.3)*	*4/13 (30.8)*	*694/1,335 (52.0)*	*149/227 (65.6)*	1,554/2,154 *(72.1)*	*1,177/1,428 (82.4)*	***<0.0001***

CV HX = cardiovascular history; GP = gastroprotectant (>91% PPI).

* Sysadoa, paracetamol, opioids; ** *P-*values denotes differences among groups overall; *** nsNSAIDS + GP data express the number of patients and % of patients with nsNSAIDs prescription that also take a gastroprotective agent. Also, COX-2 + GP data express the number of patients and % of patients with COX-2 selective inhibitors prescriptions that also take a gastroprotective agent.

Also, patients at high GI risk and no increased CV risk are recommended to take COX-2-selective NSAIDs plus PPI [12-16]; within this group, 61.8% of patients (3,521/5,697) were treated with COX-2-selective NSAIDs alone or nsNSAIDs plus PPI.

When all figures are combined, (Table 2), 51.03% (8,406/16,470) of the overall population of OA patients are being prescribed medications that were either not recommended or contraindicated, according to current guidelines and understanding.

## Discussion

Current guidelines and recommendations issued by regulatory agencies [11-16] for patients with GI and CV risk factors require NSAIDs and have introduced an unavoidable complexity in the clinical decision-making process. A recent report has shown that 86% of OA patients seen by rheumatologists are at increased GI risk and that almost half of them are at high CV risk [17]. The current study, carried out at primary care and orthopedist sites, reached a similar conclusion.

Whether recommendations issued are being followed or implemented in clinical practice is generally unknown. This study has shown that in over half of the OA population examined, the prescription of NSAIDs did not follow current guidelines or recommendations. The most critical areas where the recommendations were not being followed or were over-looked, were in patients with both high GI and CV history and in those with a high GI risk alone. In the first group, over 74% of cases received prescriptions of either non-selective NSAIDs or COX-2-selective NSAIDs. In the second group, 49% received non-selective NSAIDs and a PPI instead of no NSAID therapy or a COX-2-selective NSAID and a PPI. Naproxen was seldom prescribed overall or for those with CV risk, despite being pointed out as the safest NSAID for those with CV events [21]. However, other recommendations seem to be well adopted and followed in clinical practice. For example, our study showed high rates of PPI co-prescription with nsNSAIDs to patients with increased GI risk. These rates increased in parallel with higher levels of GI risk. Similar trends, although with lower rates of prescription, were observed with COX-2-selective NSAIDs alone. However, half of patients with low GI risk and no CV history were still treated with nsNSAIDs plus a PPI or a COX-2-selective NSAID, which are not recommended by current guidelines.

Overall, the data suggest that although some of the recommendations are being followed, others are not; especially those concerning the assessment of CV risk, which seems far from being implemented in routine clinical practice. These data contrast with some other reports at the specialist level (for example, rheumatologists), who seem to comply better with existing guidelines [17,22].

Increased blood pressure is another frequent side effect of NSAID therapy. Patients with hypertension should be monitored when receiving NSAIDs and these drugs should be avoided when patients suffer from uncontrolled hypertension. As shown in this study, over half of the OA study population received antihypertensive therapy and one-fourth had uncontrolled hypertension. An overwhelming majority of these patients with uncontrolled hypertension were receiving NSAIDs, including etoricoxib [11]. This suggests another area for improvement in clinical practice.

This study had both strengths and limitations. The strengths include: the cross-sectional approach for the collection of data in a single day for all patients; and the size of the study carried out at the primary and orthopedist care sites where OA patients are more commonly treated and prescribed therapy. Other strengths were the wide geographical distribution within the country and the fact that the data agreed with recent reports. This supports the value of the study [17]. Another important aspect of the study is that it highlights the existing problem in patients that require NSAID treatment. It reveals the necessity of guideline implementation strategies to improve clinical practice. Limitations include the fact that we had to rely on data reported by the investigators, based on recorded charts, although the consistency of the data across investigators and the multiple sites of the study reduced the impact of this limitation. Also, data on uncontrolled hypertension were based on blood pressure readings obtained during the clinical visit and confirmed with values previously recorded in patient charts when available, which may have overestimated the incidence of hypertension. However, the CV risk probably was underestimated, because it was based on the history of CV events; whereas had CV scores been available, the percentage of patients at high CV risk might have been higher [17]. However, we did not use those scores in order to simplify the study due to the size of the surveyed population. Finally, one may question whether the data obtained in patients from one country may apply to other countries. Whereas this may be true, it is possible that the validity of the conclusions may apply especially to other European countries due to similarities among health systems and the similar strategies and guidelines issued by different European scientific societies under the same regulatory body (the EMA). However, the high rates of PPI prescription found in Spain may be different from those observed in other European countries [23], which may even increase the gap of appropriate prescriptions for these patients.

## Conclusions

This study provides valuable information on the high prevalence of both GI and CV risk in OA patients who receive NSAID therapy. It also reports the high proportion of NSAID prescriptions that are not considered appropriate according to recommendations given by professional scientific societies and regulatory agencies. These data must serve not only to increase awareness of the limitations and difficulties on the translation of these recommendations into clinical practice, but also stimulate the creation of strategies or tools to increase the appropriate therapy for OA patients at primary care and orthopedist levels. These strategies may include not only educational programs but electronic tools [16], online flags and so on, that may suggest the appropriate therapy for the individual patient.

## Abbreviations

ASA: acetyl salicylic acid; COX-2: cyclooxigenase-2; CV: cardiovascular; EMA: European Medicines Agency; FDA: Food and Drug Administration; GI: gastrointestinal; NSAID: non-steroidal anti-inflammatory drugs; nsNSAID: non-selective non-steroidal anti-inflammatory drugs; OA: osteoarthritis; PPI: proton pump inhibitor; SYSADOAs: symptomatic slow acting drugs for osteoarthritis.

## Competing interests

Dr Lanas did not receive any compensation for conducting this study. Dr Lanas has received research support for investigator-initiated projects from Pfizer and AstraZeneca. He is advisor for, or has participated in, studies conducted by AstraZeneca, Pfizer, Bayer and Nicox. Beatriz Armada is currently employed by Pfizer Inc. and owns Pfizer stock. The other authors have not declared any conflict of interest

## Authors' contributions

AL revised the study design and requested the analysis to be performed. He carried out the interpretation of data and drafted the original and successive versions of the article. GGT and AOA were active investigators recruiting patients, and were involved in the interpretation of the data and in the critical and intellectual revision of the manuscript. BA had the idea to develop this study and was in charge of the coordination of the study. She also contributed to interpretation of data and contributed to the drafting of the manuscript. All authors had full access to the data and have given final approval of the version to be published. The guarantor of the article is AL

## Pre-publication history

The pre-publication history for this paper can be accessed here:

http://www.biomedcentral.com/1741-7015/9/38/prepub
